# Community Structure of Labyrinthulomycetes Protists in *Zostera marina* Seagrass Beds of Northern China

**DOI:** 10.3390/microorganisms13112420

**Published:** 2025-10-22

**Authors:** Yibo Fu, Tianle Chu, Xinlong An, Yike He, Chen Dai, Shibo Li, Yining Gu, Zhaoge Guo, Yingbo Duan, Qiuzhen Wang

**Affiliations:** 1 Ocean College, Hebei Agricultural University, Qinhuangdao 066000, China; 2 Marine Ecological Restoration and Smart Ocean Engineering Research Center of Hebei Province, Qinhuangdao 066000, China; 3 Marine Geological Resources Survey Center of Hebei Province, Qinhuangdao 066000, China

**Keywords:** Labyrinthulomycetes, seagrass bed, *Oblongichytrium*, *Stellarchytrium*, *Labyrinthula*, habitat environment

## Abstract

Labyrinthulomycetes protists play important roles in organic matter decomposition and nutrient cycling in marine ecosystems. To better understand their distribution and potential ecological functions in Caofeidian seagrass beds of the Bohai Sea, we conducted high-throughput sequencing of samples collected from multiple habitats, including leaves (L), rhizosphere (R), sediments (S), and seawater (W). Our results revealed distinct habitat-specific patterns of community composition. *Oblongichytrium* and *Stellarchytrium* were dominant in certain samples, exhibiting clear differences across stations. *Oblongichytrium* showed particularly high abundance in leaf and seawater samples, likely reflecting the availability of particulate and dissolved substrates enriched by seagrass beds. In the rhizosphere, *Sicyoidochytrium*, *Stellarchytrium* and *Labyrinthula* were enriched, whereas unclassified Labyrinthulomycetes and Thraustochytriaceae lineages prevailed in seawater and specific leaf samples. Notably, a substantial proportion of sequences corresponded to unclassified lineages, potentially representing uncultured “seagrass-associated” taxa. Compared with previous reports, our study revealed both a significantly higher abundance of *Stellarchytrium* and a remarkably greater proportion of unclassified lineages, suggesting unique features of Labyrinthulomycetes communities in the Caofeidian seagrass ecosystem. These findings provide new insights into the ecological roles of Labyrinthulomycetes in seagrass beds and offer an important reference for future taxonomic and functional studies of this group.

## 1. Introduction

Seagrass meadows are typical blue carbon ecosystems that store organic carbon in sediments in the long term while supporting biodiversity, improving water quality and mitigating acidification [1]. Hydrodynamically, seagrass leaves and rhizome networks attenuate wave energy, stabilize sediments and reduce erosion, thereby providing nature-based coastal protection and climate mitigation benefits [2,3]. However, under the dual pressures of climate warming and anthropogenic disturbances, seagrass meadows face risks of carbon stock loss and declines in ecosystem service functions.

Microorganisms play a critical role as indicators of seagrass ecosystem health. The seagrass microbiome is taxonomically and functionally diverse, with significant differences in biomass and community composition across distinct microhabitats such as leaves, roots, sediments and surrounding seawater [4,5,6]. Clear partitioning is observed between the rhizosphere and the phyllosphere, with microhabitat effects outweighing seasonal and host-species influences on community structure [7,8]. Previous studies have demonstrated that microbial compositions vary significantly among microenvironments such as the phyllosphere, rhizosphere and sediments, while functionally they often act synergistically to facilitate carbon, nitrogen and sulfur cycling [9,10]. Therefore, when investigating the microbial distribution in seagrass ecosystems, it is necessary to collect samples from different plant compartments to reveal their ecological differentiation. Moreover, the temporal and spatial dynamics of seagrass distribution can influence the structure and function of the associated microbial communities [11]. For instance, long-term environmental changes in the Bohai Sea and other northern coastal regions of China have caused significant fluctuations in seagrass habitat areas, potentially reshaping the characteristics of their associated microbiota. Consequently, distinguishing between different seagrass compartments and adjacent seawater and sediment samples in the sampling design enables a systematic analysis of the composition and distribution patterns of the associated microbial communities.

As key decomposers, members of the Labyrinthulomycetes contribute to the degradation of algae, phytoplankton and detrital material in marine and coastal ecosystems, thereby accelerating organic matter mineralization and nutrient cycling [12]. Their biomass in coastal waters may exceed that of bacterial populations, highlighting their potential ecological importance [13]. Labyrinthulomycetes are heterotrophic protists characterized by the production of ectoplasmic nets, which enable them to attach to substrates, secrete hydrolytic enzymes such as cellulases, amylases and proteases, and assimilate nutrients, thus facilitating organic carbon recycling in marine ecosystems [14,15].

Labyrinthulomycetes are widely distributed across marine ecosystems. They are particularly abundant and diverse in estuaries, lagoons and magrove habitats. These organisms occur as saprobes or epiphytes on detritus and biofilms and also establish commensalistic or opportunistic parasitic interactions with seagrasses, algae and invertebrates [16]. Their community composition is strongly influenced by dissolved organic matter, nutrient availability, salinity gradients and host distribution. For example, in the Pearl River Estuary, Labyrinthulomycetes abundance increases markedly in organic-rich and nutrient-enriched water masses [17]. High-throughput sequencing has revealed undescribed lineages and elevated β-diversity in coastal samples [18]. *Labyrinthula* is widely distributed across the Pacific and Atlantic coasts of North America, displaying strong geographic and host-specific signatures. Mangrove rhizospheres and decaying leaf surfaces represent “hotspots” of thraustochytrid abundance with clear seasonality (peaking in the post-monsoon period) [19]. Natural mangrove habitats generally host higher abundances than artificial plantations, and such environments provide abundant substrates and attachment surfaces [19]. Labyrinthulomycetes have also been recorded in offshore oligotrophic zones, deep-sea waters and sediments [13,20,21]. Although primarily marine, a few genera and species of Labyrinthulomycetes occur in freshwater environments, yet their phylogeny and ecology remain poorly understood [22]. Estuarine mixing zones may serve as transient habitats and dispersal corridors [22]. In addition, thraustochytrids are commonly found in seawater, mangrove detritus and macroalgae and may parasitize mollusks, though their role in mangrove ecosystems remains underexplored.

Labyrinthulomycetes comprise two major groups: thraustochytrids and labyrinthulids. Labyrinthulids display greater morphological diversity (fusiform, spherical, etc.) and form extensive ectoplasmic nets enveloping the cell body, enabling gliding motility. They are often associated with live algae, seagrasses and diseased algal tissues. Globally, Labyrinthulomycetes are diverse and widespread, including many uncultured and undescribed lineages from deep-sea and anoxic environments [23]. Genomic studies further indicate substantial functional divergence across genera and strains, suggesting niche differentiation [24].

Through the secretion of various hydrolytic enzymes such as cellulases, amylases and proteases, Labyrinthulomycetes participate in the decomposition of organic matter and nutrient cycling [13,25]. Furthermore, some studies have begun to reveal the potential role of Labyrinthulomycetes in marine microbial food webs. They may act as consumers of detritus and as prey for higher trophic organisms [20,25]. Certain species of Labyrinthulomycetes may be involved in the production of polyunsaturated fatty acids, which are important for marine food webs and human nutrition.

Despite the ecological significance of Labyrinthulomycetes in marine ecosystems, their roles in seagrass meadows remain poorly studied. To explore the diversity of Labyrinthulomycetes in seagrass habitats and assess their distribution across distinct ecological niches, we employed culture-independent high-throughput sequencing to characterize community composition in seagrass leaves (L), roots (R), surrounding seawater (W), and sediments (S). Considering that in situ Labyrinthulomycete cells are typically smaller than 5 μm, seawater samples were pre-filtered through a 5 μm membrane to remove larger particles, and the filtrate was subsequently collected on a 0.22 μm membrane to retain microbial cells. These findings offer valuable insights into the ecological health assessment of seagrass meadows and may guide future strategies for their sustainable utilization.

## 2. Materials and Methods

### 2.1. Sample Collection

**Figure 1:**
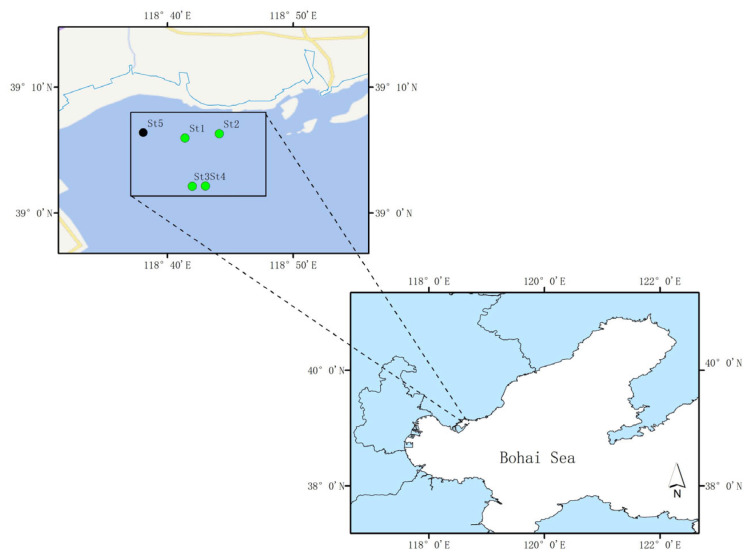
Sampling station.

The study area was located in the *Zostera marina* seagrass beds of Caofeidian, Hebei Province, Northern China (Figure 1). In recent years, this region has undergone large-scale ecological restoration, resulting in a substantial increase in seagrass coverage and improvement of the ecological environment. On 28 September 2022, five sampling stations were selected, consistent with those in [26]. Samples included seawater (W1, W2, W3 and W4), sediments (S1, S2, S3 and S4), seagrass leaves (L1, L3 and L4), and roots (R1, R3 and R4) collected from four seagrass stations (St1, St2, St3, and St4), as well as seawater (W5) from one non-seagrass control station (St5).

### 2.2. Sample Pretreatment

Seagrass samples (0.09 m^2^ per sample) were collected with a shovel and placed into sterile sealed bags. Sediment samples (~5 cm depth) were collected with a vibrating drill and transferred into sterile Erlenmeyer flasks. Seawater samples above the seagrass meadow were collected into 2 L sterile glass bottles. All samples were transported to the laboratory on ice under dark conditions and stored overnight at 4 °C until DNA extraction was performed on the following day.

### 2.3. DNA Extration

Sample pretreatment was carried out before DNA extraction [10,26]. Seagrass leaves and roots were rinsed with sterile seawater, dissected with sterile scalpels, and cut into 4 cm fragments. Several fragments were placed into 50 mL sterile conical tubes containing 30 mL sterile seawater, sonicated three times for 12 s at a frequency of 42 kHz, and filtered sequentially through 5 μm and 0.22 μm membranes. The 0.22 μm filters were subjected to DNA extraction using the Omega Water DNA Kit (Omega Bio-tek, Inc., Norcross, GA, USA). Seawater samples were filtered in the same way, and the 0.22 μm membranes were used for DNA extraction with the same kit. For sediments, 0.3 g of surface material was used for DNA extraction with the Omega Soil DNA Kit (Omega Bio-tek, Inc., Norcross, GA, USA). All DNA extracts were stored at −20 °C.

### 2.4. High-Throughput Sequencing and Statistical Analysis

High-throughput sequencing of the Labyrinthulomycetes 18S rRNA gene was performed on the Illumina NovaSeq platform and analyzed by LC-Bio (Hangzhou, China). PCR amplification of total DNA was conducted using the primer set LABY-A (5′-GGGATCGAAGATGATTAG-3′) and LABY-Y (5′-CWCRAACTTCCTTCCGGT-3′) [27,28]. PCR conditions were as follows: initial denaturation at 95 °C for 15 min, 31 cycles of denaturation at 94 °C for 30 s, annealing at 50 °C for 1.5 min, extension at 72 °C for 1.5 min, followed by a final extension at 72 °C for 10 min [27,28]. PCR products were checked by 2% agarose gel electrophoresis. No specific amplification bands were obtained for any sample from St2, root samples from St3 and all sediment samples across stations, even after attempts with modified PCR conditions and increased DNA template amounts. Consequently, only PCR products that were successfully amplified were purified using AMPure XT beads s (Beckman Coulter Genomics, Danvers, MA, USA) and subjected to sequencing.

Paired-end sequencing (2 × 250 bp) was conducted on the NovaSeq 6000 platform. Raw reads were merged with FLASH, quality-trimmed with fqtrim, and denoised using QIIME 2, version 2019.7, to generate amplicon sequence variants (ASVs). To address potential non-specific amplifications and artifacts, stringent quality control measures including chimera removal were implemented during the bioinformatics processing. α-diversity indices including observed species, Shannon index, Chao1 and Pielou’s evenness, were calculated in QIIME 2 to assess species diversity and complexity within samples. Taxonomic annotation of Labyrinthulomycetes was conducted by querying the NCBI NT database. This was performed via BLASTn alignment with stringent filtering parameters to ensure accuracy, including a minimum sequence identity (min_ident) of 90%, a minimum query coverage (min_cov) of 80%, and a maximum E-value (max_evalue) of 1 × 10^−5^. One-way ANOVA of α-diversity indices across different sample types was conducted using OriginPro 2016. Clustering heatmaps and correlation networks were generated using OmicStudio tools version 3.6 “https://www.omicstudio.cn (accessed on 8 October 2025)”, while abundance circos plots were created using the online tool of Majorbio Cloud Platform “https://cloud.majorbio.com/ (accessed on 9 October 2025)”.

## 3. Results

### 3.1. Alpha Diversity

**Figure 2:**
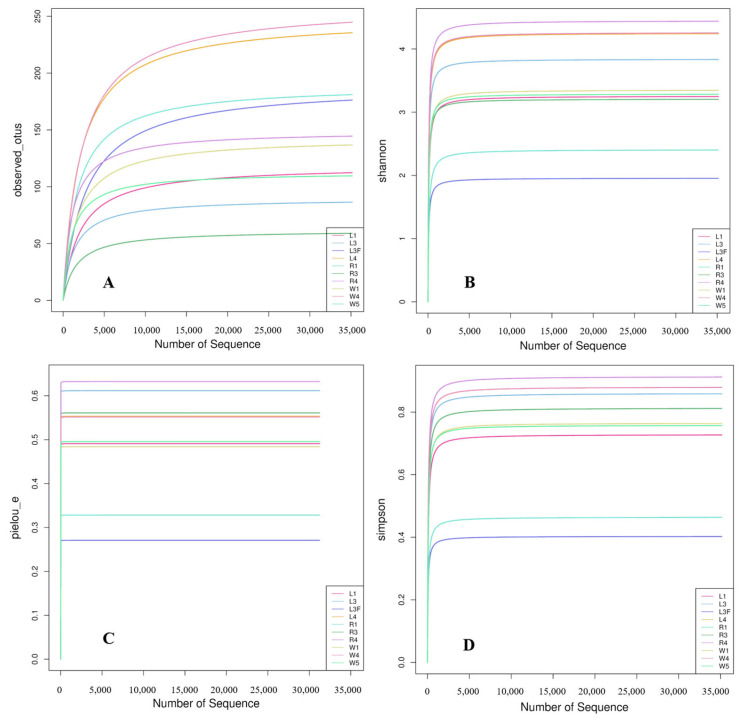
Rarefaction curves. The variation in observed species (**A**), Shannon index (**B**), Pielou’s evenness index (**C**), and Simpson index (**D**) with increased number of sequences. In the sample names, numbers indicate stations and letters indicate sample types. L1, L3 and L4 represent seagrass leaf samples; L3F represents leaf surface attachments; R1, R3, and R4 represent root samples; and W1, W4, and W5 represent seawater samples.

**Figure 3:**
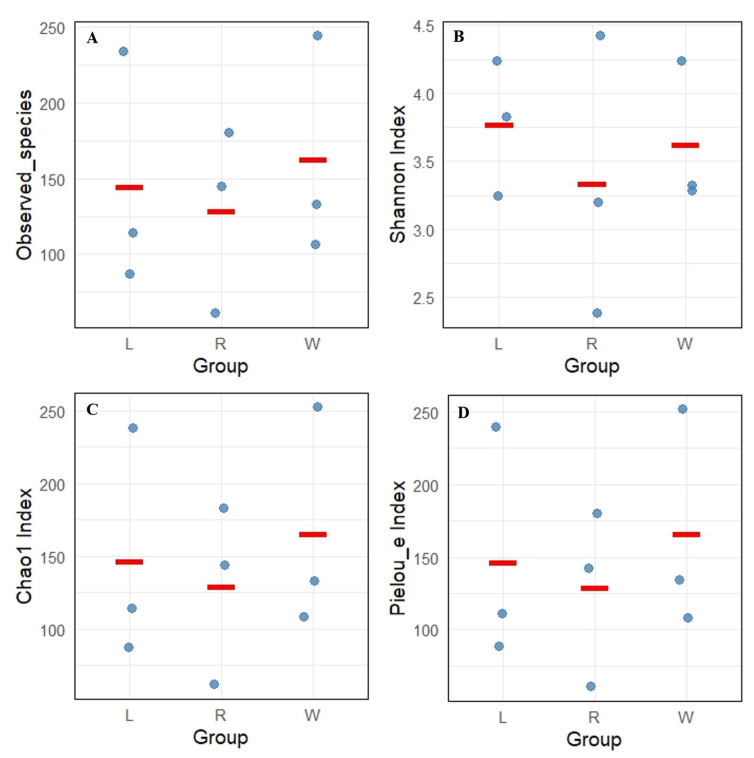
Alpha diversity index of Labyrinthulomycota communities in *Z. marina* seagrass beds, including (**A**) observed species, (**B**) Shannon index, (**C**) Simpson index, and (**D**) Pielou’s evenness index. L represents leaves; R represents roots; W represents seawater. Each dot represents the diversity index value of an individual sample, and the red line indicates the median of the data within each group.

A total of 832 ASV feature sequences of the Labyrinthulomycetes 18S rRNA gene were identified in the Caofeidian seagrass beds. Rarefaction curves of the observed species, Shannon index, Pielou’s evenness index and Simpson index indicated that both sample size and sequencing depth were sufficient (Figure 2). Both the Observed Species and Chao1 indices reflected species richness. As shown in Figure 3A,C, seawater samples (W group) exhibited the highest mean values of Observed Species and Chao1 index, indicating the greatest richness. Leaf samples (L group) ranked second, whereas root samples (R group) showed the lowest mean values for both indices, suggesting the lowest richness in the rhizosphere. The Shannon index was relatively high in L and W groups (Figure 3B), indicating higher diversity in leaf and seawater samples, with no significant difference between them (ANOVA, *p* < 0.05). In contrast, the R group exhibited the lowest Shannon index, reflecting lower diversity, which may be associated with specific environmental conditions and spatial heterogeneity. The Shannon index values were more consistent across L and W groups but more variable in the R group, suggesting uneven diversity in the rhizosphere. Pielou’s evenness index was the highest in the W group (Figure 3D), indicating the most even distribution of species in seawater, followed by the L group, while the R group exhibited the lowest evenness. In addition, no significant differences were observed among different sample types in the alpha diversity of Labyrinthulomycota communities in *Zostera marina* seagrass beds (*p* < 0.05).

### 3.2. Labyrinthulomycetes Communities in Caofeidian Seagrass Beds

**Figure 4:**
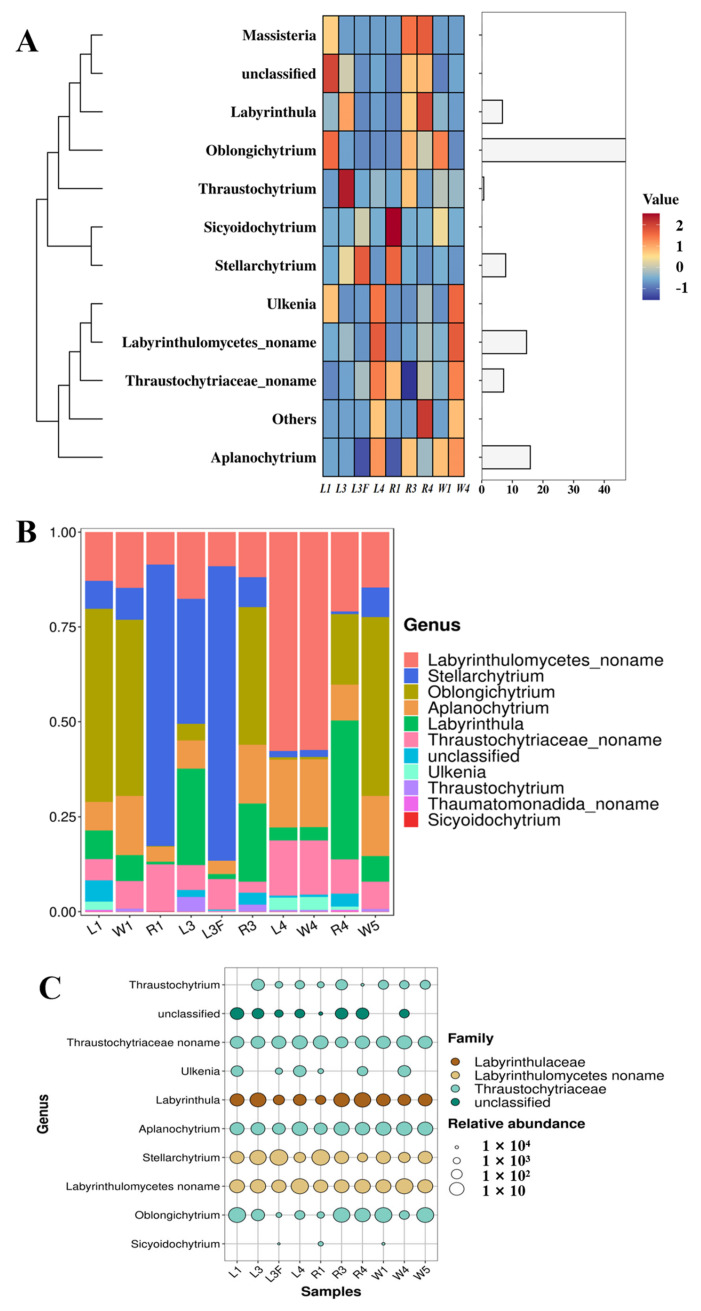
Labyrinthulomycetes Communities in Caofeidian Seagrass Beds. (**A**) The abundance ratio of different genera in the study area; The clustering on the left reflects the similarity in distribution patterns of specific labyrinthulomycete taxa across different seagrass-associated sample types; The length of the column on the right represents the proportion of a specific genus among all the collected samples. (**B**) The community composition of each sampling station at the horizontal level. (**C**) Bubble map using the top 10 genera in abundance. The “noname” annotations are a direct result of our BLASTn analysis against the NCBI NT database.

Among the top 10 most abundant Labyrinthulomycetes taxa in Caofeidian seagrass beds, the genus *Oblongichytrium* showed the highest relative abundance, followed by *Aplanochytrium*, *Stellarchytrium* and *Labyrinthula* (Figure 4A,B). Overall, the composition of Labyrinthulomycetes in seagrass leaves and seawater at the same station was relatively similar. For example, at the nearshore station St1, both leaf and seawater samples were dominated by *Oblongichytrium*, followed by Labyrinthulomycetes noname (i.e., unclassified taxa), *Aplanochytrium* and *Stellarchytrium* (Figure 4B). However, distinct differences were observed among stations. For instance, at the offshore station St3, both leaf and seawater samples were dominated by Labyrinthulomycetes noname, followed by *Aplanochytrium* and Thraustochytriaceae noname. Notably, at St1, unidentified Labyrinthulomycetes accounted for over 70% of the total community, highlighting the substantial role of unclassified taxa in coastal ecosystems. Moreover, *Stellarchytrium* was a dominant genus across multiple stations and sample types (R1, L3F and L3), suggesting its broad ecological distribution and adaptive capacity in diverse environments. As shown in Figure 4C, *Aplanochytrium*, *Stellarchytrium*, Thraustochytriaceae noname, and Labyrinthulomycetes noname were widely distributed across different sample types, whereas genera such as *Thraustochytrium*, *Ulkenia*, *Oblongichytrium* and *Sicyoidochytrium* showed large variation in relative abundance among samples, with some genera, e.g., *Sicyoidochytrium*, absent from most samples.

### 3.3. Differential Distribution Patterns of Labyrinthulomycetes Across Sample Types

**Figure 5:**
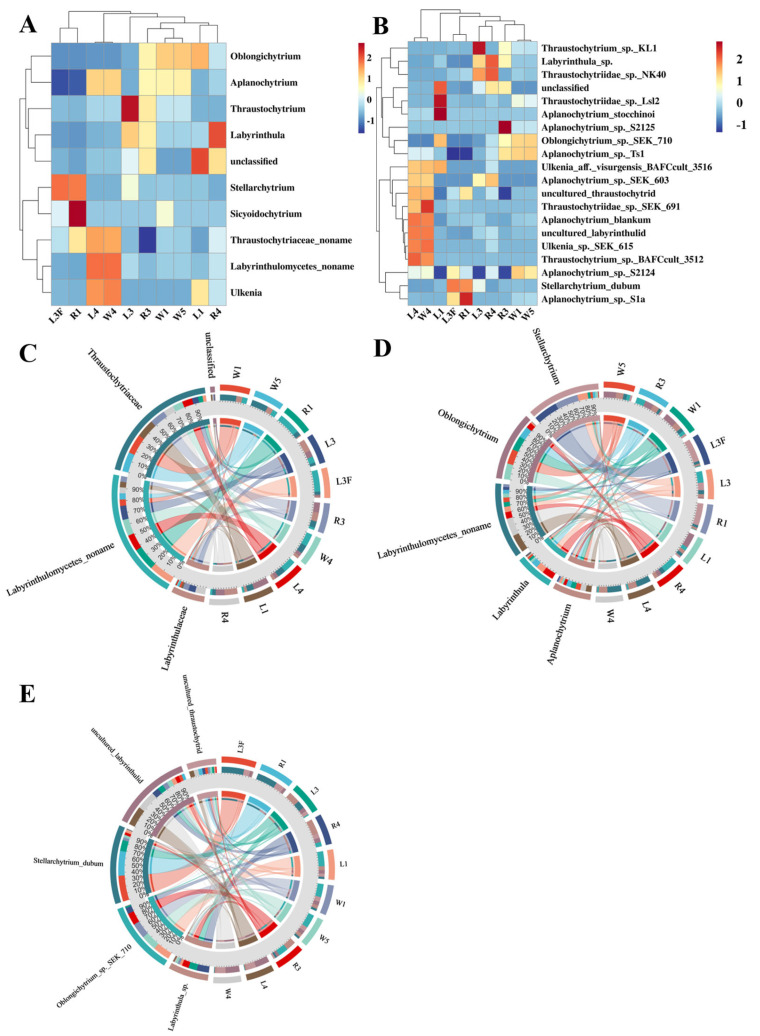
Distribution patterns of Labyrinthulomycetes across different sample types. (**A**) Clustering heatmap using the top 10 genera in abundance of Labyrinthulomycetes. (**B**) Clustering heatmap using the top 30 species in abundance of Labyrinthulomycetes. The circle charts are used to represent the distribution of the top5 most abundant Labyrinthulomycetes at the level of families (**C**), genura (**D**) and species (**E**); Each sector in different colour represents a taxon, and its size corresponds to the relative abundance proportion of that taxon within all Labyrinthulomycetes.

At the genus level, clustering analysis showed that the different sample types (R4, L4 and W4) from station St4 clustered together, whereas samples from other stations formed another distinct branch (Figure 5A,B). Within St4, the leaf sample L4 and the seawater sample W4 were more closely related, while both were slightly distant from the root sample R4. The dominant genera shared by L4 and W4 included *Thraustochytrium*, Thraustochytriaceae noname, *Ulkenia*, *Aplanochytrium* and Labyrinthulomycetes noname. For stations other than St4, no distinct clustering patterns were observed. The circos plots at the family, genus and species levels illustrated the relationships between samples and the top five abundant taxa, reflecting both the dominant taxa within each sample and their distribution proportions across different groups (Figure 5C–E). At the family level, Thraustochytriaceae dominated the Labyrinthulomycetes communities in Caofeidian seagrass beds, being present in nearly all samples, with the highest proportions in L1, W1 and W5, followed by R3, R4, W4, and L4, but much lower in R1, L3, and L3F. The second most abundant family was Labyrinthulaceae, mainly distributed in R3, R4 and L3, with only minor proportions in other samples. In addition, Labyrinthulomycetes noname groups were significantly enriched in R1, L3F, L3, L4 and W4, possibly suggesting enhanced organic matter degradation activities (e.g., humus-rich environments), and highlighting these habitats as potential sources for the discovery of novel Labyrinthulomycetes species. At the genus level, the top five taxa were Labyrinthulomycetes noname, *Stellarchytrium*, *Oblongichytrium*, *Aplanochytrium*, and *Labyrinthula*, with Labyrinthulomycetes noname being especially abundant in L4 and W4. At the species level, the top five taxa were uncultured labyrinthulid, *Stellarchytrium dubium*, *Oblongichytrium* sp. SEK710, *Labyrinthula* sp. and uncultured thraustochytrid.

### 3.4. The Relationship Among Labyrinthulomycete Protists

**Figure 6:**
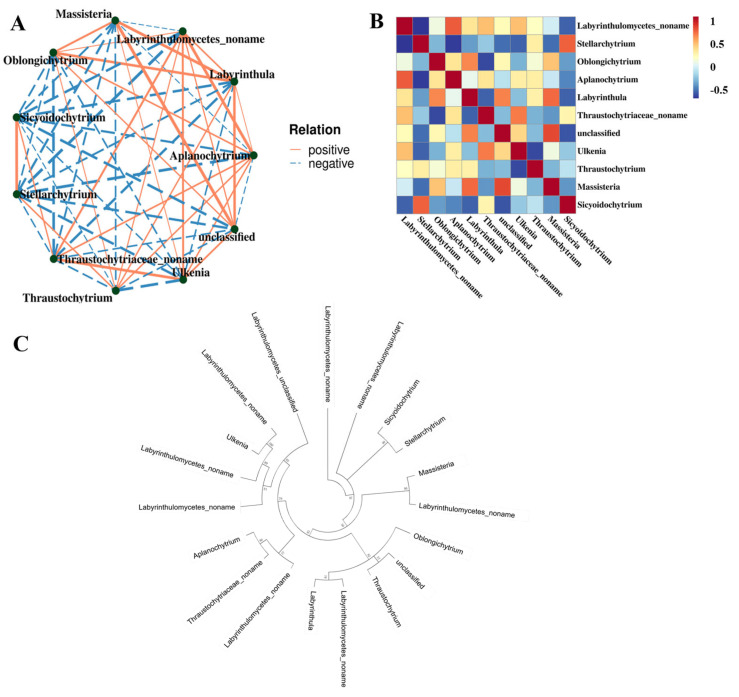
The relationship among the Labyrinthulomycetes protists at the genera level. (**A**) Correlation network diagram. Each node represented a dominant genus, with node color indicating its taxonomic affiliation at the phylum level; Edges represented correlations between genera, where only relationships with |rho| > 0.4 were displayed; The thickness of the edges indicated the strength of correlation; Thicker lines denoted stronger correlations, while thinner lines represented weaker correlations; Solid lines indicated positive correlations, whereas dashed lines indicated negative correlations; Node size corresponded to the number of associations with other taxa, with larger nodes representing genera that were more interconnected. (**B**) Spearman heatmap. (**C**) Neighbor-joining phylogenetic tree based on the amplicon sequence variant (ASV) sequences.

To explore the interactions among different Labyrinthulomycetes taxa, a Spearman correlation-based network analysis was performed using the top 11 genera in terms of relative abundance (Figure 6A). Most genera that were phylogenetically close also showed positive correlations in the Spearman network (Figure 6B,C), suggesting potential mutualistic or symbiotic interactions, such as between *Ulkenia* and Labyrinthulomycetes noname. However, exceptions were observed. Although *Stellarchytrium* and *Sicyoidochytrium* are phylogenetically related, they showed a negative correlation in the network. This result indicates potential competition between the two genera, possibly driven by overlapping nutrient requirements.

## 4. Discussion

### 4.1. Dominant Distribution Patterns of Labyrinthulomycetes in Seagrass Beds

In the Caofeidian seagrass bed, protists of the class Labyrinthulomycetes exhibited a pronounced ecological niche differentiation. It was observed that *Oblongichytrium* and *Stellarchytrium* dominated in certain samples, a pattern distinct from their distribution in other Marine habitats [29]. Previous studies have reported that *Aplanochytrium* was the most abundant genus in coastal and shelf waters [13,20], while *Aurantiochytrium* dominates in oceanic deep waters [20]. By contrast, *Oblongichytrium* is usually present at low abundances and shows a patchy distribution pattern [13,20,21]. However, in Caofeidian seagrass bed, *Oblongichytrium* displayed relatively high abundances in both leaf and water samples, which may be attributed to the enriched and specialized particulate or dissolved substrates provided by the seagrass habitat. Seagrass leaves and roots continuously secrete a mucilaginous layer enriched in hemicellulose, pectin and phenolic polysaccharides. Compared with pelagic particulate organic matter (POM) or marine snow, these substrates exhibit lower cellulose crystallinity and higher phenolic content, which are more suitable for utilization by groups such as *Oblongichytrium* with specialized enzymatic repertoires for degrading complex polysaccharides [25]. This could explain the localized “blooms” of *Oblongichytrium* within the seagrass bed. In contrast, the sediment environment is often hypoxic or even anoxic, with highly reducing chemical conditions, e.g., elevated sulfide concentrations, which are unfavorable for the survival and reproduction of aerobic Labyrinthulomycetes [30]. Moreover, a substantial proportion ~25% of the community was composed of unclassified Thraustochytriaceae and Labyrinthulomycetes lineages, which may represent uncultured “seagrass-associated” species. This highlights the uniqueness of the seagrass bed ecosystem and its potential as a reservoir of novel Labyrinthulomycetes taxa.

### 4.2. Community Variability in Labyrinthulomycetes Across Ecological Niches

The community composition of Labyrinthulomycetes varied markedly among different ecological niches (leaf, root, and seawater) within the Caofeidian seagrass bed. In leaf-associated samples, *Oblongichytrium* and Labyrinthulomycetes_noname were dominant, particularly in L1 and L4 samples, respectively, where they accounted for over 50% of the community. This suggests that these taxa may play important roles in organic matter degradation on seagrass leaf surfaces [12]. In contrast, the L3 sample was characterized by higher relative abundances of *Stellarchytrium* and *Labyrinthula*. This indicates that even if they are all seagrass leaf samples, the microenvironment heterogeneity of leaves at different stations will form different community structures. This variability may be closely related to dissolved organic matter exudation, co-attached microbial communities and local hydrodynamic conditions on seagrass surfaces.

The community composition in root samples was significantly different from that of leaves. In R1, *Stellarchytrium* was overwhelmingly dominant, whereas in R3, the proportions of *Oblongichytrium*, *Labyrinthula* and *Aplanochytrium* were notably higher. Root-associated sediments are enriched in organic matter and root exudates, which likely provide selective conditions favoring certain taxa. For instance, *Labyrinthula* is known to establish either pathogenic or symbiotic associations with seagrass hosts. *Labyrinthula zosterae* has been identified as the causal agent of seagrass wasting disease [31]. Therefore, the enrichment of *Labyrinthula* in the root-associated habitat may reflect complex ecological interactions with host root tissues.

In comparison, seawater samples displayed yet another pattern. In W1 and W5, *Oblongichytrium* accounted for relatively high abundances, whereas in W4, Labyrinthulomycetes_noname dominated, suggesting that a substantial fraction of the waterborne community consists of unclassified taxa. These findings underscore the importance of high-throughput sequencing in uncovering “cryptic diversity” [32], and further indicate that the Bohai Sea harbors abundant but poorly studied Labyrinthulomycetes lineages.

### 4.3. Regional Distributional Differences in Labyrinthulomycetes Communities

Compared with previous studies, results of the present study revealed both common patterns and regional differences. In terms of commonality, several genera of the family Thraustochytriaceae, e.g., *Oblongichytrium*, *Aurantiochytrium* and *Schizochytrium* were consistently detected in seawater and seagrass bed microhabitats [13,33], with some taxa being well known for their capacity to decompose organic matter and synthesize highly unsaturated fatty acids [25,34]. *Labyrinthula* represents another important lineage that may act as a saprotrophic decomposer in organic matter cycling, while also functioning as a potential pathogen [31,35], which is consistent with the present findings.

Regional differences were observed in three main aspects. First, *Stellarchytrium* exhibited relatively high abundances in the leaf and rhizosphere samples of this study, particularly dominating R1 and L3 samples, whereas reports of this genus have been rare in tropical or temperate ecosystems [36]. This suggests that *Stellarchytrium* may occupy a unique ecological niche in the cold-temperate Bohai Sea or gain a competitive advantage under specific environmental conditions. Second, in seawater samples, unclassified lineages such as Labyrinthulomycetes_noname and Thraustochytriaceae_noname accounted for a substantial proportion, which differs from patterns reported in traditional culture-based or reference-sequence-dependent studies. This not only reflects the distinctive community composition of Labyrinthulomycetes in the Bohai Sea but also highlights the current gaps in their taxonomic framework, suggesting that novel genera or species may be uncovered from these unclassified clades in the future [32]. Third, compared with tropical or temperate marine regions, the typical lipid-producing genera *Schizochytrium* and *Aurantiochytrium* did not emerge as dominant groups in this study, likely due to differences in water temperature, nutrient conditions or host-associated factors in northern waters.

In addition, *Stellarchytrium* was reported to be first established as a new genus by FioRito et al. [36] during their investigation of Asteroid-associated Labyrinthulomycetes. Its distinct morphological and phylogenetic characteristics clearly separate it from closely related taxa such as *Aplanochytrium* and *Oblongichytrium*. Subsequent taxonomic studies incorporated it into the systematic framework of Labyrinthulomycetes, underscoring its independent status in community diversity [29,30]. More recently, environmental amplicon surveys revealed that “*Stellarchytrium*-like” lineages can exhibit seasonal dominance in certain marine environments, suggesting that this group may play a unique ecological role in coastal organic matter degradation and biogeochemical cycling [13]. Future research should incorporate a broader spectrum of environmental parameters, including nutrient concentrations, oxygen levels, and sediment characteristics, in conjunction with metagenomic analyses. Such an integrative approach would not only help to elucidate the factors driving the distribution and abundance of Labyrinthulomycetes but also enhance our understanding of their ecological significance within marine ecosystems.

## 5. Conclusions

This study analyzed the communities of Labyrinthulomycetes protists in the Caofeidian seagrass bed and revealed their distribution patterns across different microhabitats (leaf, rhizosphere and seawater). Results showed that *Oblongichytrium* and *Stellarchytrium* were dominant in certain samples, displaying distinct distribution patterns. *Oblongichytrium* exhibited high abundance in leaf and seawater samples, which may be associated with the specific substrates provided by seagrass beds. In addition, a considerable number of unclassified Labyrinthulomycetes lineages, accounting for 25% of the total abundance, were detected, which may represent yet-uncultured “seagrass-associated” Labyrinthulomycetes. Obvious differences in community composition were observed among the microhabitats and sampling stations. Oblongichytrium and Labyrinthulomycetes_noname dominated the leaves. *Stellarchytrium* and *Labyrinthula* were more abundant in the rhizosphere, while Labyrinthulomycetes_noname and Thraustochytriaceae_noname were dominant in seawater. Compared with previous studies, this study found relatively high abundances of *Stellarchytrium* in both leaf and rhizosphere samples, as well as a notably higher proportion of unclassified lineages, suggesting a unique composition of Labyrinthulomycetes communities in the Bohai Sea. Overall, the Labyrinthulomycetes communities in the Caofeidian seagrass bed exhibited obvious structural differences among microhabitats and a high degree of “cryptic diversity.” These findings provide new insights into the ecological roles of Labyrinthulomycetes in seagrass beds and offer an important reference for future research on the taxonomy and functional ecology of this group. Future studies should further investigate the taxonomic positions and ecological functions of the unclassified lineages.

## Data Availability

The original data presented in the study are openly available at https://www.ncbi.nlm.nih.gov/, accession number: PRJNA1042847.
